# Conformational changes in Chikungunya virus E2 protein upon heparan sulfate receptor binding explain mechanism of E2–E1 dissociation during viral entry

**DOI:** 10.1042/BSR20191077

**Published:** 2019-06-28

**Authors:** Bibekananda Sahoo, Tirumala Kumar Chowdary

**Affiliations:** School of Biological Sciences, National Institute of Science Education and Research (NISER), Homi Bhabha National Institute (HBNI), Bhubaneswar 752050, India

**Keywords:** alphavirus receptor, Chikungunya virus entry, E2 conformational changes, E2-E1 dissociation, receptor-binding, viral entry

## Abstract

Receptor binding is the first step in viral cell entry. In enveloped virus cell entry, viral and host membrane fusion follows receptor binding. Viral surface receptor-binding protein associates with membrane fusion protein and masks its structure, to prevent pre-mature fusion activity. Dissociation of receptor-binding protein from fusion protein is an essential step before membrane fusion. Mechanism of receptor binding leading to dissociation of receptor binding and fusion protein is poorly understood in alphaviruses. Chikungunya virus (CHIKV), an alphavirus, re-emerged as a global pathogen in recent past. CHIKV surface envelope proteins, E2 and E1, function as receptor binding and fusion protein, respectively. Site of heparan sulfate (HS) receptor binding on E2–E1 heterodimer and its effect on E2–E1 heterodimer conformation is not known. Using molecular docking, we mapped HS binding to a positively charged pocket on E2 that is structurally conserved in alphaviruses. Based on our results from docking and sequence analysis, we identified a novel HS-binding sequence motif in E2. Purified E2 binds to heparin and HS specifically through charge interactions. Binding affinity of E2 to HS is comparable with other known HS–protein interactions (*K*_d_ ∼ 1.8 μM). Mutation of charged residues in the predicted HS-binding motif of E2 to alanine resulted in reduction of HS binding. Molecular dynamics (MD) simulations on E2, after docking HS, predicted allosteric domain movements. Fluorescence spectroscopy, far-UV circular dichroism spectroscopy, fluorescence resonance energy transfer experiments on HS-bound E2 corroborate our findings from MD simulations. We propose a mechanism where receptor-binding results in allosteric domain movements in E2, explaining E2–E1 dissociation.

## Introduction

Chikungunya virus (CHIKV) is an aedes mosquito-transmitted re-emerging alphavirus that causes chikungunya fever, a debilitating disease [1]. Since its re-emergence in epidemic proportions, in several South East Asian countries during 2006–2010 [2,3], CHIKV has spread to Europe and America, where the virus was never seen before [4].

CHIKV is an enveloped virus (has a protective lipid bilayer on surface). Similar to other enveloped viruses, CHIKV enters cells in a sequential, two-step process: (i) virus binding to cell surface receptor, followed by endocytosis and (ii) fusion of viral envelope with endocytic vesicle membrane that leads to opening of a fusion pore. Nucleocapsid escape into cytoplasm follows membrane fusion [1,5,6]. The viral surface envelope-anchored proteins, E2 and E1, perform receptor binding and membrane fusion functions, respectively. Membrane fusion by E1 fusion protein follows a universally conserved mechanism: fusion protein is ‘masked’ by receptor-binding protein (E2 in alphaviruses) on viral surface, and only upon receptor binding, fusion protein is unmasked and triggered (by endosomal acidic pH in viruses that enter through endocytosis) to undergo conformational changes required for membrane fusion activity [1,5,6]. Thus, receptor-binding protein regulates pre-mature activation of fusion protein by structurally masking it. Dissociation of receptor-binding protein from fusion protein is an essential step in the mechanism of membrane fusion in viral entry.

In CHIKV envelope proteins, E2 and E1 are anchored into viral membrane through short transmembrane segments. A third protein, E3, is on viral surface through its interaction with E2 [1,7]. Role of E3 in entry is not known. In CHIKV-infected cells, E3-E2-E1 is made as a single polypeptide, and proteolytically processed into individual proteins as they mature through secretory pathway. E3-E2 and E1 exist as a heterodimer on virion surface [8–10]. Henceforth, E3-E2–E1 heterodimer will be referred to as E2-E1.

**Figure 1:**
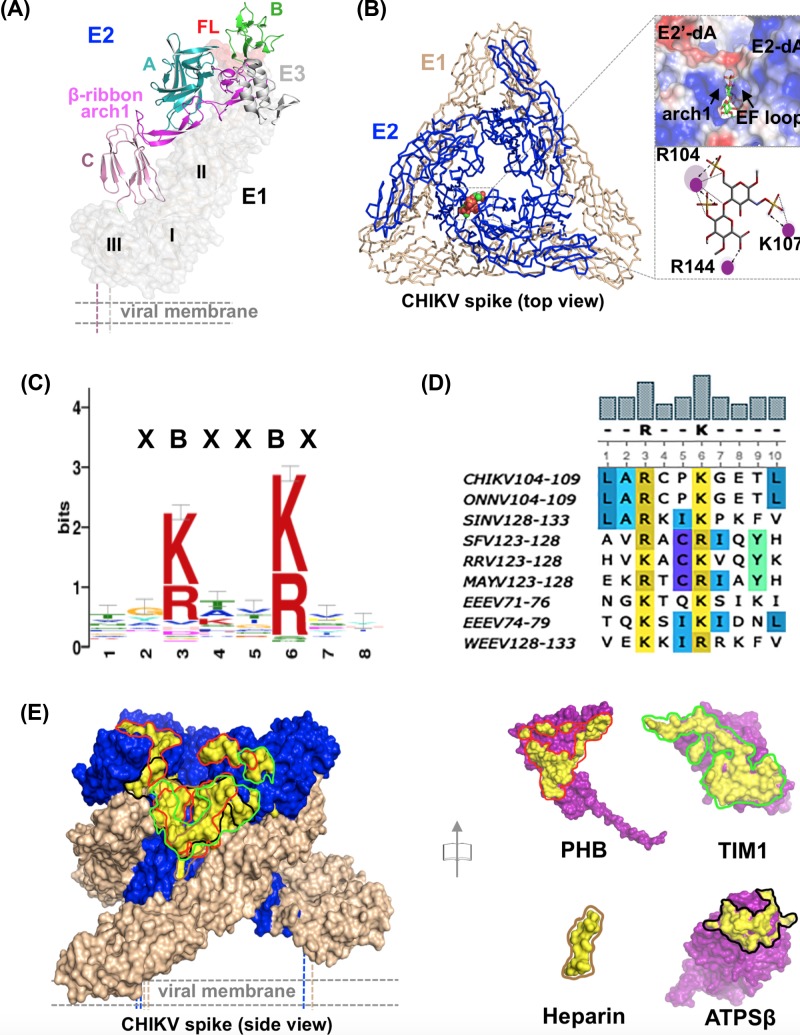
Mapping of HS and protein receptor-binding sites on CHIKV E2-E1 trimeric structure (**A**) Domain organization of E2–E1 heterodimer (PDB: 3N42). (**B**) Left – heparan sulfate (HS) (spheres) docked pose of E2-E1 spike (ribbon representation). Upper right – zoomed in view of HS-binding pocket shown as electrostatic potential surface. E2-dA and E2′-dA are domain A from two adjacent protomers in trimeric spike. Arch 1 and EF loop are indicated by arrows, HS is in stick representation. Lower right – 2D interaction plot, generated using Discovery studio visualizer, BIOVIA, shows H-bonds (as ….) and electrostatic interactions (as ←---→) between E2 residues and HS. (**C**) Consensus alphavirus E2-HS binding motif logo. Level of positional conservation is represented as bit score on y-axis. (**D**) XBXXBX motif in E2 sequence of few alphaviruses. MAYV - Mayaro virus; ONNV - Onyong-nyong virus; RRV - Ross River virus; WEEV - Western equine encephalitis virus. (**E**) Binding site of PHB (red outline), T-cell immunoglobulin mucin domain 1 (TIM1) (green outline), heparin (brown outline) and ATPS-β (black outline) mapped on spike structure. Each receptor structure, with E2-binding interface outlined, is shown in open book orientation to the right.

Ectodomains of E2 and E1 have predominantly β-sheet secondary structure. E2 and E1 have three domain architecture: from viral membrane proximal to distal – domains III, I and II in E1; C, A and B in E2 (refer Figure 1A) [8]. In E2, a β-ribbon connector runs alongside domain A. E3 interacts with E2 through β-ribbon connector. E2 and E1 interaction spans through all three domains, with most interactions between domain C of E2 and domain II of E1. An internal fusion loop (FL), which inserts and anchors into host membrane during fusion, is present at tip of the domain II of E1. FL is masked by domain A and B of E2, forming a ‘clamp’ like structure [8]. FL of E1 can be unmasked only when E2 A and B domains move away from E1 domain II.

On CHIKV surface (and in other alphaviruses as well), E2–E1 heterodimers follow the icosahedral symmetry of the viral capsid, because of E2 C-terminal tail interaction with the capsid protein. As a result, three E2–E1 heterodimers assemble to form a spike-like structure, with E1 at the periphery and E2 at the center (Figure 1B) [8]. At the end of membrane fusion, as interpreted from acidic pH structure of E1 from an alphavirus [11], E1 becomes a homotrimer. As imaginable, and proposed earlier [8–10], reorganization of E2-E1 to E1 homotrimers is possible only if E2 dissociates from E1, and gets displaced from center of the spike. What triggers E2–E1 dissociation? Receptor binding has been proposed as a triggering event for E2–E1 dissociation. Conformational changes in viral surface proteins, upon receptor binding, were proposed as the earliest events before alphavirus entry [12,13]. Apart from this, endosomal acidic pH is also shown to lead to conformational changes in E2–E1 heterodimer and dissociation [10,14,15]. However, how receptor binding to E2 alters its conformation to trigger its dissociation from E1 is not known.

CHIKV shows broad cell and tissue tropism. CHIKV can infect keratinocytes, fibroblasts, muscle stem cells, monocytes and macrophages [16]. Though a cognate receptor of CHIKV is not known, several cell surface proteins, prohibitin [17], TIM1—a phosphatidylserine receptor [18], matrix remodelling associated protein (MXRA8) [19], ATP synthase β-subunit (ATPS-β, in insect cells) [20] and Glycosaminoglycans (GAGs) are reported as binding partners. GAGs such as HS are present on many cell types, and hence can be used as universal receptor for alphaviruses. CHIKV, Sindbis virus (SINV), Semiliki Forest virus (SFV) and Eastern Equine Encephalitis virus (EEEV) can use HS and heparin as a receptor for cell entry [21–24]. Though several proteins and heparan sulfate have been reported as CHIKV receptors, their binding site on E2 is not thoroughly characterized.

In this study, using molecular docking and biochemical approaches, we characterized HS-receptor binding site on CHIKV E2, and studied conformational changes in E2 upon HS binding. We found that HS binds to a structurally conserved, positively charged pocket on E2. Interestingly, HS-binding sequence in E2 does not match to other consensus heparin/HS-binding motifs. By sequence analysis, we identified a new HS-binding sequence motif (XBXXBX, where B is a basic residue), that is present in all alphaviruses. Using purified E2 protein, we show that E2–HS interaction is specific. Mutation of charged residues (Arg104 and Lys107) in predicted CHIKV E2 HS-binding pocket to alanine resulted in significant loss of HS binding. Molecular dynamics (MD) simulation on HS-docked E2 structure revealed dramatic changes in tertiary structure of E2 conformation upon HS binding. Results from intrinsic tryptophan fluorescence, far-UV circular dichroism (CD) spectroscopy and fluorescence resonance energy transfer (FRET) studies on E2 in absence or presence of HS support our MD simulation predictions. Based on our results, we propose a model to explain mechanism of E2–E1 dissociation after receptor binding: binding of receptor on domain A results in allosteric movement of domain C of E2, pushing E1 away from trimeric spike central axis.

## Materials and methods

### Molecular docking

We used PyMol plugin version of Autodock Vina [25] to perform docking studies. We used heparan sulphate disaccharide (ZINC id. 85551958) and CHIKV E2-E1 trimeric structure extracted as a single .pdb file from PDB:2XFB as ligand and receptor, respectively. We prepared receptor and ligand pdb files by removing water molecules, adding hydrogens and charges to polar residues. Using Autogrid program, assigned appropriate grid dimensions along XYZ directions with grid spacing of 0.375 Å. We performed a global docking search using default parameters. We chose docking pose with least ΔG value for analysis.

For protein–protein docking using ClusPro server [26], we used trimeric spike extracted from PDB:3J2W [27] as receptor and Prohibitin (PDB: 1LU7) or TIM1 (PDB: 2OR8) or ATPS-β (5FIL) as ligands. For protein–heparin docking, we used the default heparin tetrasaccharide (PDB: 3QMK) from the advanced protein–heparin docking module of ClusPro. We performed a global search over the whole protein surface, with program default heparin conformational flexibility parameters. Best docking pose is selected from the top-ranked clusters obtained with balanced mode. Balanced energy term has weight coefficients values as E = 0.40E_rep_ ± 0.40E_attr_ + 600E_elec_ + 1E_DARS_, in the equation E = w1E_rep_ + w2E_attr_ + w3E_elec_ + w4E_DARS_, where E_rep_ and E_attr_ denote the repulsive and attractive contributions to van der Waals interaction energy, and E_elec_ is an electrostatic energy term, as implemented in ClusPro protein-protein and protein–heparin docking algorithm. E_DARS_ is a pairwise structure-based potential constructed by the decoys as the reference state (DARS) approach. It primarily represents desolvation contributions to free energy change due to removal of water molecules from the interaction interface.

### HS-binding motif search on alphavirus E2 sequences

We searched for HS-binding motif in E2 sequences from different alphaviruses using Multiple Em for Motif Elicitation server [28]. First, we searched for canonical heparin/HS-binding motif (HBD) in E2 sequences. Later, we refined our search to discover a consensus HBD in E2 sequences, combining knowledge from our docking studies with published data on other alphavirus mutations that effect E2–HS interactions. We chose sequence stretches of 11 amino acids from E2 sequences, where the centric residue is reported in the literature to effect viral entry through HS binding. We included 16 alphavirus E2 sequences for motif search. Altogether, we compared 110 input sequences for presence of a consensus by using the following parameters: site distribution – one occurrence per sequence, number of motifs to find = 1, background model = 0-order, motif width = 6–10 residues.

### Cloning, expression and purification of E2, E3E2 and E3E2 mutants

PCR amplicons of full-length E2 and E1-coding regions from CHIKV LR2006 isolate are a kind donation from Prof. Scott C. Weaver, UTMB, Galveston, Texas. We prepared E3-coding DNA fragment, using overlapping short oligonucleotides in an assembly PCR method, following a published protocol [29]. We cloned E2 or E3E2 ectodomain coding regions into a bacterial expression vector, pET24b. All clones were sequence verified and used for expression in BL21(DE3) strain of *Escherichia coli*. Overexpressed E2 and E3E2 proteins were in insoluble fraction of cell lysate. We solubilized the proteins from inclusion bodies using 20 mM Tris-HCl pH 8.0 buffer containing 150 mM NaCl and denaturant 8 M Urea. Phenyl methyl sulfonyl fluoride (purchased from Sigma-Aldrich, St. Louis, MO, U.S.A.) (100 μM) was added to lysate as protease inhibitor. Proteins were purified from urea-solubilized fractions using Ni-NTA His-tag affinity chromatography. Purity of proteins was assessed by SDS-PAGE. Purified proteins were refolded by exchanging into TN buffer containing 1 mM EDTA (TNE), and concentrated, using ultrafiltration method. Protein concentration estimation was done using absorbance at 280 nm and molar extinction coefficients.

Mutations in predicted HS-binding motif on E2 (E3E2 - R104A, K107A and R104A+K107A) were generated by site-directed mutagenesis approach using primer-extension method. We also created serine to cysteine mutations, S154C and S296C in β-ribbon connector and domain C of E2, respectively, for coupling a FRET pair of fluorophores. All mutant constructs were sequence verified before expression and purification. Expression and purification of mutant proteins was done exactly the same way as that used for the wild-type protein. Mutant proteins were characterized for structure and function (HS binding) in the same way as wild-type protein.

### Heparin or HS binding assays

To characterize E2 binding with heparin, we used heparin sepharose (HiTrap heparin HP, GE Healthcare Life Sciences, U.S.A.) affinity chromatography as a binding assay. We applied 10 μg of recombinant E2 protein onto a heparin column in TNE buffer (conc. of NaCl is 150 mM). After an intermittent wash step with TNE buffer, we eluted the bound protein with buffer containing increasing concentration of NaCl (equal volumes of 0.3 M, 0.6 M and 1 M NaCl). Aliquots of equal volume from each fraction (load, wash and NaCl elution) were analysed on SDS-PAGE followed by Coomassie Brilliant Blue R-250 (CBB) staining. BSA was used as a control to assess non-specific binding of proteins to heparin. The amount of protein eluted with 300 mM or higher NaCl concentration is taken as a measure of specific binding of protein to heparin through electrostatic interactions. To prove the specificity of binding, we performed a competition assay by pre-incubating E2 with HS (heparan sulfate sodium salt from bovine kidney purchased from Sigma–Aldrich, U.S.A.) before loading on to heparin column.

We also used E3E2 or E3E2 Arg/Lys to Ala mutants in HS-binding assay to measure the extent of binding. To calculate the fraction of protein bound to heparin column specifically, we performed the binding assays as described above, except that the elution was done in a single fraction with 1 M NaCl. Extent of protein-heparin binding is represented as percent of protein loaded (fraction bound), by quantifying the band intensities using imageJ software. Mean ± SD values of three independent experiments is presented in a bar graph and compared for statistical significance of differences in one-way ANOVA analysis using GraphPad Prism 7.0a software (GraphPad Software, U.S.A.).

### Microscale thermophoresis experiment

Purified E3E2 protein was fluorescently (NT-547, Amine-reactive Green dye supplied with Monolith™ NT Protein Labelling kit GREEN-NHS) labelled using N-hydroxysuccinimide labelling protocol. The protein concentration (0.2 mg/ml, 5 μM) was kept constant, while the concentration of the non-labelled HS varied from 0.03 to 15 μM. After 30-min incubation at RT, the complex samples were loaded into Microscale thermophoresis (MST) standard coated capillaries and analysis was performed using the Monolith™ NT.115 instrument with 40% MST power. Raw data were analyzed with the software MO. Affinity Analysis (version 2.2.7; NanoTemper), and the final curve fitting was done with the data points (each data point is represented as the mean value of three independent measurements) using GraphPad prism software.

### MD simulations

We carried out MD simulations, energy minimization and trajectory analyses on the HS-docked E2 complex structure with the GROMACS 5.1.4 package [30] using the GROMOS96 (43a1) forcefield. We performed the MD run on a 12 core Intel Xeon processor workstation. We used explicit SPC water molecules in all simulations. Briefly, we added water molecules around the solute molecules within a cubic water box, using periodic boundary conditions. We inserted counter ions for system neutralization. The system was energy minimized using steepest descent algorithm. LINCS algorithm was applied to constrain the bonds. Solvent density was set to the value corresponding to 1 atm at 300 K. Solute and solvent were coupled independently to a modified Berendson bath at 300 K, with the coupling time constant of 0.1 ps. Electrostatic interactions were calculated with the PME method, using non-bonded cut offs of 1.4 nm for Coulomb and 1.4 nm for van der Waals. The MD integration time step was 2 fs. The output coordinates were updated every 10 ps. The system was equilibrated twice, first for 100 ps under NVT, followed by 100 ps under NPT condition prior to final MD run. The production MD run was for 10 ns at 300 K.

PyMol was used for structure visualization, making figures and distance measurements between domain center of mass.

### Circular dichroism and fluorescence spectroscopy

Far-UV CD and intrinsic tryptophan fluorescence spectra were recorded with protein alone and with protein-HS mixture (at 1:50 molar ratio). Samples were incubated at room temperature for 30 min before recording the spectra. Protein was used at 0.2 mg/ml concentration in TNE buffer.

The far-UV CD spectra were recorded using a 0.1 cm path length quartz cuvette. Mean residue ellipticity (θmrw) values against wavelength spectra presented in the figures are average of three spectral accumulations each, and blank corrected. The percentage of secondary structure content of the protein was predicted from the far-UV CD spectra, using K2D3 software available at http://cbdm-01.zdv.uni-mainz.de/∼andrade/k2d3/.

Fluorescence emission spectra were recorded using a 1 cm cuvette and excitation wavelength was set to 295 nm. Average of three spectral accumulations is presented in the figures, after blank correction.

### FRET experiments

Cysteines were introduced, replacing serine residues at 154 and 296 position, in E3E2 to enable labelling with a FRET fluorophore pair. The mutant protein was reacted with Ellman’s reagent and number of free cysteines available were estimated from Ellman’s reagent absorbance at 412 nm. After ensuring that only introduced cysteines are available for labelling, E3E2 S154C + S296C protein was labelled with the FRET pair, Alexa Fluor™ 488 and 594 C5 Maleimide (Thermo Fisher Scientific, U.S.A.) through cysteine thiol-reactive maleimide chemistry, following protocol prescribed by the manufacturer. Protein and fluorophore maleimide were used at a molar ratio of 1:20 in the labelling reaction. Reaction mixture, in TNE buffer, was incubated at RT for 2 h in dark. The labelled protein was separated from free dyes using Sephadex G-25 in PD-10 desalting columns (GE Healthcare Life Sciences, U.S.A.).

Fluorescence emission spectra were recorded with excitation wavelength set to 493 nm (λ_max_ abs. for Alexa Fluor™ 488) and emission was recorded with wavelength range 500–700 nm. Spectra were collected either with the labelled protein alone or with the labelled protein incubated with HS (with 50 molar excess to protein) for 30 min at RT. Excitation and emission slit widths were set to 5 nm.

## Results

### HS binds in a positively charged pocket between domain A and arch 1 of β-ribbon connector of E2

To predict HS-binding site(s) on E2-E1 trimeric spike surface, we performed molecular docking of HS disaccharide structure on to E2-E1 trimeric spike structure. Best docking pose, as assessed by least Gibbs free energy score (ΔG = −7.6 kCal/mol) is used to analyze for HS-binding site on E2-E1 trimer. Figures 1A, B show crystal structure of E3-E2-E1, and the docked pose of HS on trimeric spike, respectively. HS is bound on the apex, towards interior of the spike, in a positively charged pocket between two juxtaposed A domains from two E2 protomers in the trimeric spike (Figure 1B). The predicted HS-binding site on E2 is a cleft formed between domain A and arch 1 of β-ribbon connector (Figure 1B, right top) with arginine and lysine residues lining the binding site, as expected of a HS-binding site on a protein [31]. HS is bound by charge–charge interactions between side chains of Arg104, Lys107 of E2 (Figure 1B, right lower) with sulfates from HS. Two of the binding residues Arg104 and Lys107 are part of a contiguous stretch in the sequence, and part of the loop connecting strand E and F of the domain A β-barrel. The third-binding residue, Arg144, is from arch1 of β-ribbon connector (Figure 1B).

Further, we compared HS-binding motif on E2 that we predicted from docking studies, with consensus HS-binding motifs (HBDs) reported for other HS/heparin-binding proteins. There are two consensus HBD-sequence motifs: (i) XBBBXXBX and (ii) XBBXBX, where X is any residue and B is a basic residue (arginine/lysine) [32]. Other HS-binding motif sequences, XBBBXXBBBXXBBXBX and TXXBXXTBXXXTBB (T is turn, B is a basic residue, X is a hydropathic residue), were proposed for the Von Willebrand factor and α- and β-fibroblast growth factors (αFGF, βFGF), respectively [31]. Interestingly, the HS-binding pocket sequence on E2, (AR_104_CPK_107_G), did not match to any of the known HBDs. Then, we mapped mutations in E2 (from CHIKV and other alphaviruses) that were reported in published literature [8,16] to either increase or decrease HS binding. Interestingly, most of those mutations congregated in a region close to the binding pocket that we predicted for HS. This prompted us to ask a question, does alphavirus E2 have a unique and minimal HS-binding motif? To answer this, we did motif scan with search pattern combining knowledge on mutations that effect HS binding in alphaviruses and results of our docking studies. A consensus sequence motif that emerged as HS-binding pattern in alphavirus E2 sequences is shown in (Figure 1C). Based on these results, we infer that XBXXBX is a novel HBD in alphaviruses. Sequence motifs matching to XBXXBX pattern are found in all most all alphavirus E2 sequences that we analyzed. Few alphavirus E2 HBDs are aligned and shown in Figure 1D. We also docked other reported CHIKV receptor protein structures and heparin tetrasaccharide structure on to E2 structure. Binding sites for heparin tetrasaccharide, Prohibitin, TIM1, ATPS-β also mapped to the same region on domain A and β-ribbon connector (Figure 1E), overlapping with predicted HS-binding site.

### E2 protein binds to heparin/HS through predicted HBD

**Figure 2:**
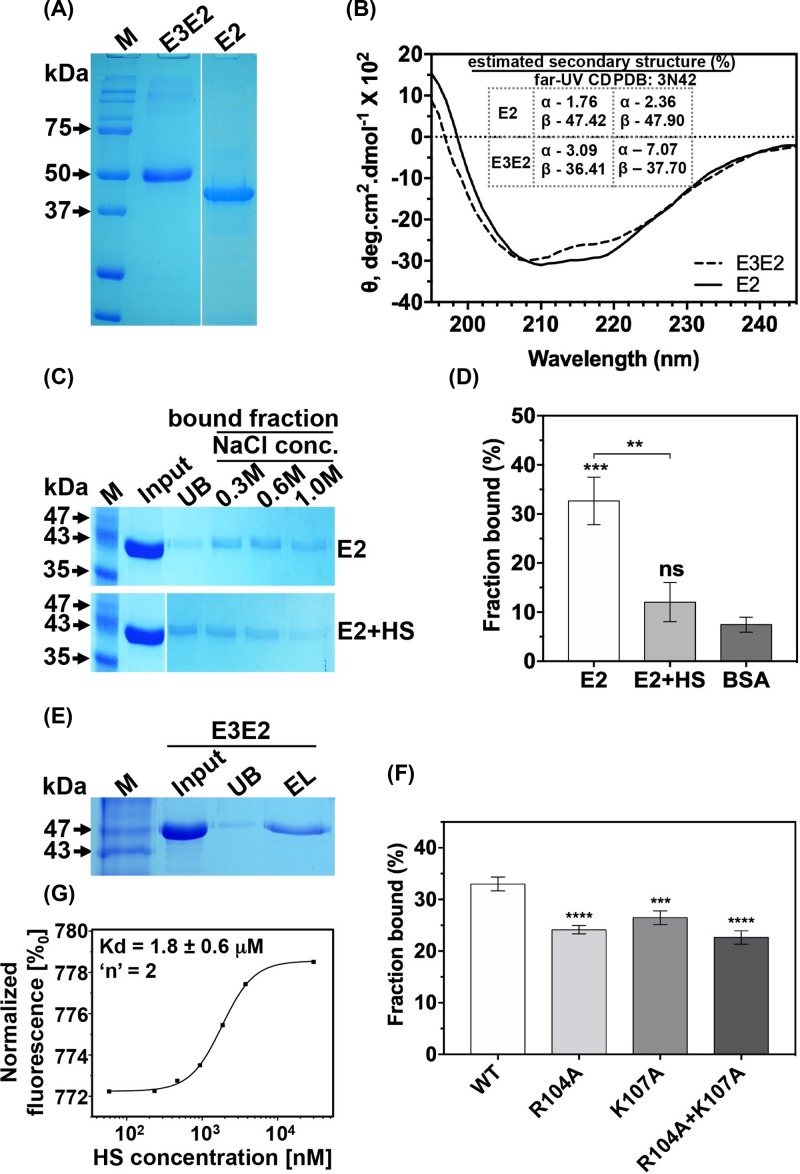
Biochemical characterization of CHIKV E2 and E3E2 protein-heparin/HS binding (**A**) SDS-PAGE analysis of purified E3E2 and E2 proteins. (**B**) Far-UV CD spectra. Inset table shows % secondary structural content estimates from far-UV CD data versus crystal structure (**C**) Elution of E2 from heparin sepharose column. E2 (top gel image) and E2 pre-incubated with HS (bottom gel image). Lane labels: M - mol. wt. marker, UB - unbound, bound fraction - protein that eluted with NaCl. (**D**) Quantification of % bound protein fraction. E2+HS: E2 pre-incubated with HS in competition assay. ** and *** represent: *P*≤0.01 and *P*≤0.001, respectively; ‘ns’: non-significance. (**E**) Elution of E3E2. Lane EL: 1 M NaCl elution fraction. (**F**) Comparison of E3E2 WT and E3E2 R104A, K107A and R104A+K107A proteins in heparin-binding assay. Mean ± SD (*n*=3) of % fraction bound to heparin is presented for each protein, with statistical differences calculated to E3E2 WT protein. *** and **** represent: *P*≤0.001 and *P*≤0.0001 level of significance, respectively. (**G**) Dose response curve for HS binding to CHIKV E3E2 with *K*_d_ value and Hill’s coefficient (*n*) value from the MST experiment.

To experimentally validate our docking results of HS-binding site on E2, and to study conformational changes in E2 upon HS binding, we expressed and purified E2 or E3E2 proteins (Figure 2A, and E2, E3 sequences given in Figure S1). In CHIKV-infected cells, E3E2 and E1 are folded together into a heterodimer. Earlier studies to understand structure of CHIKV entry proteins, expressed the proteins in insect cells [8], which produced E3E2-E1 as an irreversible complex. However, to understand conformational changes in E2 upon receptor binding, expression and purification of E2 alone is desirable. To test if the bacterially expressed E2 or E3E2 is folded in the same way, as in the E2–E1 heterodimer, we characterized its secondary structure using far-UV CD spectroscopy. Secondary structure content estimated from far-UV CD data showed 47.42% of β-sheet for E2 (Figure 2B and table inset with in). This is in perfect agreement with estimated β-sheet content (47.90%) for E2 from crystal structure (PDB: 3N42, in complex with E1 and E3) indicating that the protein we purified folded into predominantly β-sheet similar to native conformation of E2. Intrinsic tryptophan fluorescence spectroscopy on E2 showed that the protein is folded with proper tertiary structure — tryptophan fluorescence emission max. at 335 nm.

In E2–heparin-binding assays, significant amount of protein remained bound even at 300 and 600 mM of NaCl concentration (Figure 2C, top), indicating that binding is through specific, electrostatic interactions. Elution with 1 M NaCl was required to release all the bound E2 from heparin. Soluble HS, incubated with E2, prior to heparin-binding assay, significantly reduced binding of E2 to heparin sepharose (Figure 2C, bottom). This further confirms that E2 binding to heparin is specific in nature. The fraction of E2 bound to heparin is significantly higher (32.6%) than fraction of BSA bound (7.4%) to heparin (Figure 2D).

There are four regions in CHIKV E2 sequence that match to HBD pattern, XBXXBX. In purified E2 protein, all four sites may be available for HS binding. Mapping these sites onto E3-E2-E1 crystal structure revealed that two of the four sites are in a region that would be masked by E3-E2 interface on virion surface. We purified E3E2 protein, confirmed that it is folded to its native conformation (Figure 2A, B), and tested its binding to heparin. If in E2, the HBDs in the E3–E2 interface also contribute to HS binding, then E3E2 protein should have reduced binding to HS than E2. In our heparin-binding assays, E3E2 bound to heparin (Figure 2E) to the same extent (fraction bound 32.9%, Figure 2F) as E2 alone, indicating that the HBDs in E3–E2 interface do not contribute to HS binding.

From the predicted HBD on E2 (docking studies), AR_104_CPK_107_G, R104 and K107 make electrostatic interactions with sulfates of HS. We mutated these charged residues to alanine. We characterized structure of the mutants by far-UV CD and intrinsic tryptophan fluorescence, and confirmed that mutants folded in the same way as wild-type protein (Figure S2). Binding of E3E2 R104A / K107A / R104A+K107A to heparin was significantly reduced compared with wild-type protein (Figure 2F). However, mutations in HBD of domain A did not lead to complete loss of binding. There are three other predicted HBDs in CHIKV E2 sequence. As explained above, two of the other three HBDs masked in E3–E2 interface did not contribute to HS binding in our biochemical assays, as E2 and E3-E2 proteins bound to HS equally. However, one more predicted HBD in domain B would still be available for binding in E2 or E3E2. This site may be contributing to the remaining HS binding to the E3E2 HBD mutants. But, this HBD (in domain B) would not be available on virion surface for HS binding (would have been masked in E2–E1 interface on virion surface). Our docking studies used a virion surface trimeric spike structure of E2-E1, where no HS docking was seen in domain B HBD.

Further, we observed that extent of loss of HS binding to E3E2 in double mutant (R104A+K107A) is not an additive effect of each single mutant (R104A or K107A). Several earlier studies characterizing HS and other glycosaminoglycan–protein interactions have reported similar results with HS-binding site mutations. For example, a recent study by Richard et al. [33] has reported non-additive effect of single, double or triple mutations of HBD in antithrombin, on heparin binding.

We characterized strength of E3E2 binding to HS by microscale thermophoresis analysis. The estimated equilibrium dissociation constant (*K*_d_) value of 1.8 ± 0.6 μM shows that the binding affinity is weak. Our observations are in line with *K*_d_ values reported for other proteins binding to HS [34]. More than one HS-binding sites were estimated on E3E2 (*n*=2) (Figure 2G). This further supports our observations with E3E2 R104A+K107A binding to heparin, as explained in above paragraph.

Taken together, our docking predictions and comparison of heparin binding to purified E2, E3E2 or E3E2 R104A / K107A / R104A+K107A mutant proteins strongly suggest that HBD between domain A and arch 1 of β-ribbon connector is the primary-binding site for HS/heparin receptor. On virion surface trimeric spike this site (of the four HBDs on E2) would be the most accessible site for HS binding.

### HS binding to E2 results in movement of domain C in, towards E1

**Figure 3:**
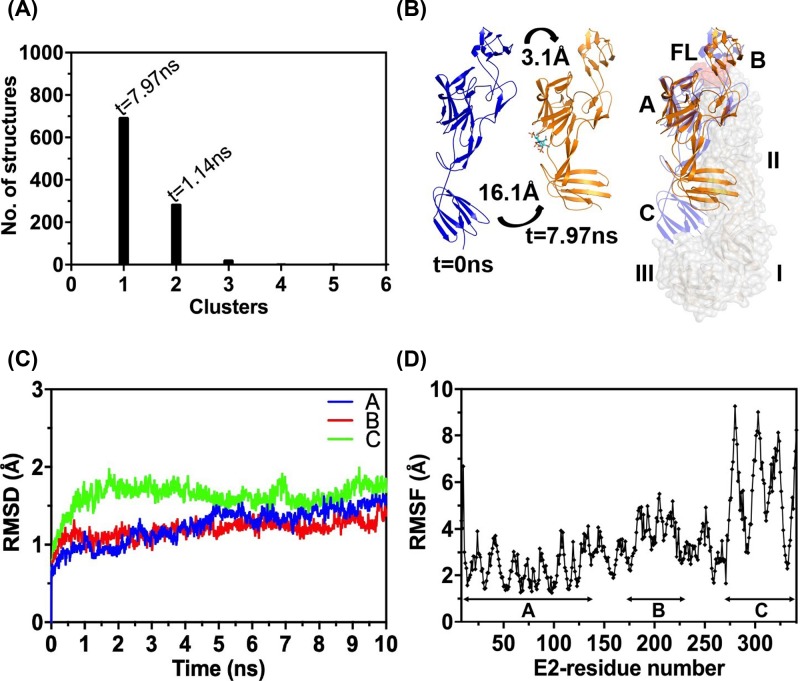
Analysis of MD simulation trajectory of E2–HS complex (**A**) Clustering analysis of the trajectory. (**B**) Left: comparison of *t* = 0 ns with *t* = 7.97 ns structure (central structure of major cluster). Curved arrows indicate movement in domain B and domain C. Right: overlay of E2–E1 heterodimer structure with *t* = 7.97 ns structure. E1 (grey, surface representation) domains represented as I, II, III; and for E2 as A, B, C. E2 (blue, cartoon representation) and E2-HS, *t* = 7.97 ns structure (orange) are aligned. HS is shown in stick representation. FL is E1 FL. (**C**) Domain wise RMSD and (**D**) RMSF analysis output.

Receptor binding on E2 is the first triggering event in alphavirus entry. However, how receptor binding leads to breaking of E2–E1 interactions and dissociation is not known. Does HS binding on E2 lead to conformational changes? To test this, we performed MD simulations on HS-docked E2 structure. Clustering analysis of simulation trajectory, with 4 Å cut-off, revealed two major clusters in a six clusters group: cluster1 with 692 and cluster 2 with 284 structures (Figure 3A). We compared HS-docked E2 structure (pre-simulation structure) with central structure of the major cluster1. From the overlay of structures it is apparent that the positions of domains B and C changed compared to their starting positions (Figure 3B). The domain B of E2 has shifted from the completely masking position over the FLs in domain II of E1 to a slightly outward position. Center of mass for domain B has moved by 3.1 Å from its position in pre-receptor-bound conformation (Figure 3B, left). Backbone RMSD analysis reflected the same (Figure 3C). Major domain movement is seen with domain C of E2—all atom RMSF analysis showed maximum deviation (Figure 3D) and center of mass moved by 16.1 Å (Figure 3B). Overlay (Figure 3B) shows that domain C moved into a place where domain II of E1 would be in the heterodimer complex. Overall secondary structure of the protein did not change upon HS binding.

**Figure 4:**
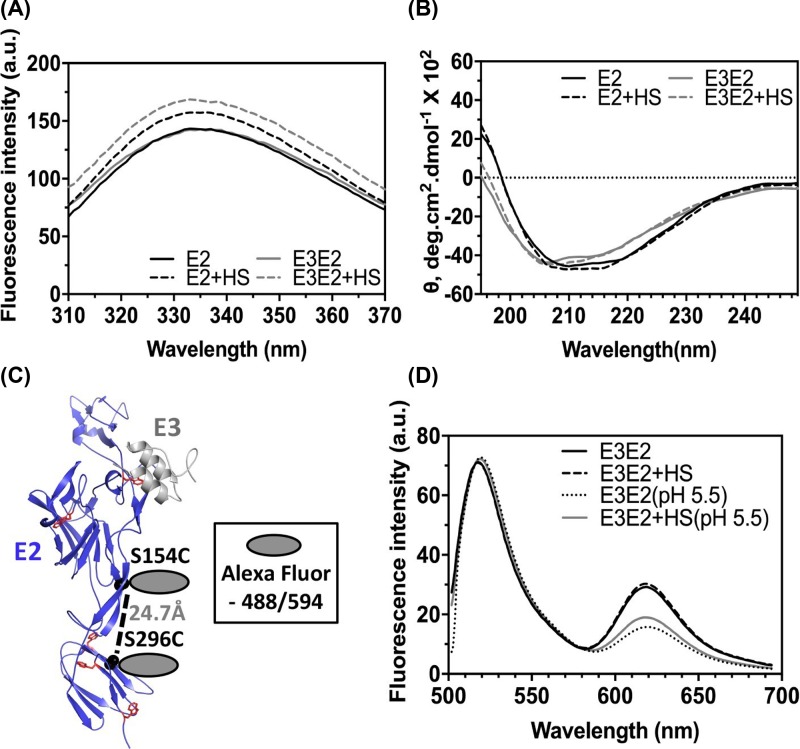
Tertiary and secondary structure analysis on HS-bound E2 and E3E2 (**A**) Intrinsic tryptophan fluorescence spectra and (**B**) Far-UV CD spectra of E2 and E3E2 in presence and absence of HS, respectively. (**C**) Structure of E2 (in cartoon representation) with Ser154 and Ser296 (shown as spheres) locations chosen for cysteine mutation are shown. C_β_ to C_β_ distance between the residues is shown. Tryptophans in E2 are shown in stick representation. (**D**) FRET data represented as fluorescence emission spectra of Alexa Fluor labelled E3E2 (either at pH 7.4 or pH 5.5), in presence or absence of HS.

Three out of five tryptophans present in E2 are located in domain C. We reasoned that, if domain C moves upon HS binding (as predicted in MD simulation), tryptophan residues (at least for those in domain C) environment is likely to change. Tryptophan fluorescence emission spectra in presence and absence of HS are shown as an overlay in Figure 4A. In presence of HS, intrinsic tryptophan fluorescence intensity increased for both E2 and E3E2, indicating that there is a change in tertiary structure upon HS binding. On the contrary, far-UV CD recorded for the proteins, in presence or absence of HS, did not show any significant changes (Figure 4B and Table 1), indicating that there are no secondary structural changes. We infer that the changes observed in tryptophan fluorescence are primarily because of bending of domain C to bring three of the five tryptophans close to β-ribbon connector.

**Table 1:** Estimated % secondary structural content in E2 and E3E2

% secondary structural content		Far-UV data (K2D3 predicted values)		Crystal structure (calculated from PDB: 3N42)	
protein (± HS)		α-helix	β-sheet	α-helix	β-sheet
E2	−HS	1.76	47.42	2.36	47.9
	+HS	2.5	47.74		
E3E2	−HS	3.09	36.41	7.07	37.70
	+HS	3.08	36.45		

To further validate, we generated a cysteine double mutant of E3E2 to introduce FRET acceptor-donor pair onto domain C and β-ribbon connector and confirmed that the mutant protein is similar to wild-type with respect to secondary structure content and tertiary structure, as deduced from intrinsic tryptophan fluorescence and far-UV CD spectroscopy (Figure S3A, B). We probed available free cysteines with Ellman’s assay, to make sure that only introduced cysteines are available for fluorophore labelling–Ellman’s reagent absorbance at 412 nm for wild-type is same as buffer blank, whereas for double cysteine mutant it increased (Table S2). Double cysteine mutants, labelled with FRET fluorophore pair bound to HS, to similar extent as wild-type protein (Figure S3C). Using the labelled E3E2 protein, we studied domain movements upon HS binding by FRET. We carefully chose places for cysteine introduction, to avoid any possible effect on HS binding to the cysteine double mutant. We measured the distance between sidechains of Ser154 and 296 (from E2 crystal structure [8]) to be 24.7 Å (Figure 4C). FÖster distance (R0) reported for the FRET pair used is 60 Å (as per manufacturer, Thermo Fisher, website). This implies that good efficiency FRET should be seen, if E3E2 folded similar to crystal structure conformation. As expected, FRET between donor and acceptor could be seen with labelled protein alone (Figure 4D). This further confirms that the purified protein has domains organized same as seen in crystal structure. However, contrary to the expectation of increased FRET after HS binding to E3E2, we did not see any significant change in acceptor or donor fluorescence emission (Figure 4D) – MD simulations predicted that domain C would move by ∼16 Å, bringing it close to β-ribbon connector. This could be because the donor and acceptor fluorophores are much closer (∼20 Å) on the protein than R0 (∼60 Å), and hence FRET is near to 100% efficiency. In this scenario, domain movements upon HS binding (as predicted in MD simulations) would not result in perceivable change in FRET. If this assumption is right, then at slightly acidic pH (5.5), where β-ribbon connector has been shown to be dissociated from domain A contacts [15], should increase the distance between FRET donor and acceptor, and thus result in reduced FRET. In fact, FRET with E3E2 in pH 5.5 buffer is reduced (Figure 4D) (lower acceptor emission and higher donor emission) compared with that at pH 7.4, indicating that our assumption about distance between the FRET pair on E3E2 at pH 7.4 is right. Change in FRET at pH 5.5 is not altered by HS addition to E3E2 (Figure 4D).

## Discussion

CHIKV has spread globally since its re-emergence in recent past. Similar to other alphaviruses, CHIKV enters cells through endocytic pathway. Viral envelope protein E2 is the receptor-binding protein. Despite several years of study, knowledge on E2–receptor interaction is very limited. Also, mechanistic details of E2-E1 action after receptor binding are not understood. In this study, using *in silico* and biochemical approaches, we characterized heparan sulfate and heparin interaction with CHIKV E2 protein. We studied changes in E2 structure upon HS binding, that can explain E2–E1 dissociation mechanism—an essential step before membrane fusion. Here, we discuss our results in the light of literature available on alphavirus E2–E1 dissociation mechanism during entry.

CHIKV [21,23,24] and several other alphaviruses such as SINV [35,36], SFV [35], RRV [37], EEEV [38] can use HS as a cell surface-binding partner for entry. Our molecular docking predicted that HS binds to a positively charged pocket on trimer of E2-E1. The pocket is between domain A and arch 1 of β-ribbon connector of E2. A structurally analogous positively charged pocket is seen in trimeric spike structures of other alphaviruses, SINV [10] and VEEV [39], as well. The predicted HS binding site on CHIKV E2 is part of EF loop of domain A. Interestingly, the EF loop is highly conserved, both in terms of sequence and structure in alphaviruses [7].

Other viral envelope proteins such as HIV gP120 [40] and Respiratory syncytial virus G [41], Dengue virus E [42] and HSV gD [43] that bind to HS have consensus HBD motifs. Alphavirus E2 sequences do not have consensus HBD motifs. We identified a novel heparin/HS-binding sequence motif pattern, XBXXBX, in alphaviruses. Sequences matching to XBXXBX motif pattern are found in all most all other alphavirus E2 sequences, and map to domain A. For example, in SINV HBD is in CD loop of domain A, projecting Arg and Lys sidechains in to the HS-binding pocket in a similar structural context as seen in CHIKV E2. Earlier studies by Voss et al. [8] mapped mutations in other alphavirus E2 sequences that effect HS binding on to CHIKV E3-E2-E1 structure, and inferred that domain A, β-ribbon connector and domain B are putative receptor-binding regions on E2. Several alphavirus neutralizing antibody escape mutations are also mapped to the same region. In another study, Kam et al. [44] mapped epitope of a CHIKV-neutralizing antibody to a region on β-ribbon connector, close to the HS-binding pocket proposed in this study. Interestingly, predicted binding regions for other protein receptors as well map to domain A or β-ribbon connector, overlapping with HS-binding region on E2 (Figure 1E). In a recent study, Zhang et al. [19] mapped MAb epitopes that block MXRA8-receptor binding to E2 domain A and domain B.

We validated our docking predictions using purified E2 or E3E2 proteins. E2 and E3E2 binds to HS and heparin specifically, though through weak electrostatic interactions (*K*_d_ = 1.8 ± 0.6 μM). Mutation of either Arg104 or Lys107 or both from predicted HS-binding motif sequence lead to significant loss of HS binding. Taken together, our docking studies and biochemical studies affirm that the charged pocket at the boundary of domain A and β-ribbon connector on E2 is the binding pocket for HS receptor.

Structures of trimeric spike from CHIKV [8,10], SINV [10], VEEV [39] show E2 at the center of the spike. Domain A and β-ribbon connector from E2 make most of the intraspike contacts. Whereas, E2 domain C and β-ribbon connector interactions with E1 domain II keep E1 in the trimeric spike periphery. In post-fusion structure, E1 is a homo-trimer. Receptor binding [8,12,13] and acidic pH of the endosome [10,14,15] were suggested as reasons for E2–E1 dissociation. It is also possible that receptor binding to E2 initiates heterodimer dissociation, which then is completed during endosome acidification. Either way, for E2–E1 dissociation to happen major inter-protein contacts between domain C of E2 and domain II of E1 have to be broken. Opening of E2 domain A and B ‘clamp’ around E1 FLs may happen subsequently or simultaneously to dissociation of domain C–domain II. To understand which of these events are triggered by receptor binding, we performed MD simulations on HS-bound E2 structure. Our MD simulation on E2-HS bound structure predicted movement of domain C, and reorganization of domain B, upon HS binding. No significant deviation in conformation is observed in domain A where HS is bound. Intrinsic tryptophan fluorescence, and FRET studies in the presence of HS also indicate movement of domain C. As imaginable, in the E2–E1 heterodimer, this movement of domain C, would sterically clash with domain II of E1, and probably push E1 away from the trimer axis. Domain B is displaced marginally away from domain A in HS-bound structure. This would open up the domain A and B clamp around E1 FL and would break a critical hydrogen bond between His226 of strand F from E2 domain B and backbone of Ala92 on E1 FL. Earlier Li et al. [15] using 3D reconstruction of SINV E2-E1 trimer crystal structure onto SINV cryo-EM map showed that domain A–B ‘clamp’ opens up, exposing the FLs of E1, as an intermediary conformational change step during entry. They also speculated domain C rearrangements as a possible mechanism for E2–E1 dissociation.

**Figure 5:**
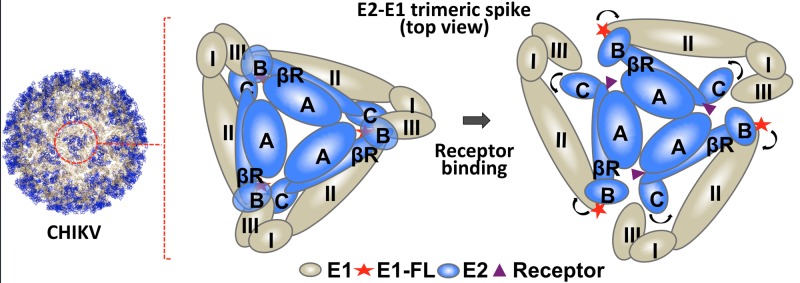
Mechanism for alphavirus E2–E1 dissociation upon receptor binding Top view of E2-E1 trimeric spike (zoomed in view from the viral surface) before (left), and after receptor binding (right). E1 domains are represented as I, II and III (wheat-coloured ovals), and E2 domains are represented as A, B and C (blue coloured ovals). Receptor (purple triangle) binding to E2 on domain A or β-ribbon connector allosterically induces domain movements in E2: E2 domain C movement pushes E1 domain II, resulting in E2–E1 dissociation. E2 domain A-B clamp opening exposes E1 FL (red star) at the tip of domain II.

Based on our observations, and interpreting our results in the light of structure information available on different alphavirus E2-E1 proteins, we propose a mechanistic model for receptor binding as a triggering event in alphavirus entry (Figure 5). We propose that positively charged groove formed between domain A and arch 1 of β-ribbon connector is a conserved HS receptor-binding region on E2 in alphaviruses. Receptor binding on domain A, allosterically triggers movement of domain C towards E1, pushing E1 away from trimeric spike. Simultaneously or subsequently, domain B moves away from domain A, opening up and exposing E1 FLs. Domain movements upon receptor binding to trigger fusion protein activation have been reported for other viruses as well. In measles virus, binding of receptor to H (the receptor-binding protein) results in movement of head domains relative to each other, to trigger fusion activity of fusion protein, F [45].

Despite several studies on receptors of alphaviruses, mechanism of receptor binding as trigger in cell entry remained enigmatic. Our study provides first clues to understand receptor binding as a regulation mechanism in alphavirus cell entry.

## Supporting information

**Supplementary Figure S1:** Sequence of CHIKV E3E2 and E2 protein used in this study. Protein sequence of E3 and E2 protein from Chikungunya virus strain LR2006_OPY1 (extracted from full-length structural poly-protein sequence, protein_id="ABD95938.1")

**Supplementary Figure S2:** Characterization of E3E2 WT and HBD mutants, E3E2 R104A, K107A and E3E2 R104A+K107A, proteins. (A) Intrinsic tryptophan fluorescence spectra of wildtype and single or double mutant proteins. (B) far-UV CD spectra of wildtype and single or double mutant proteins. (C) SDS-PAGE gel images from the heparin affinity binding experiment are shown in a comparison between E3E2 WT and both single and double Ala mutant proteins.

**Supplementary Figure S3:** Characterization of E3E2 WT and S154C+S296C mutant protein. (A) Intrinsic tryptophan fluorescence spectra of wildtype and double cysteine mutant proteins. (B) far-UV CD spectra of wildtype and double cysteine mutant proteins. (C) SDS-PAGE gel images from the heparin affinity binding experiment are shown in a comparison between E3E2 WT and the double cysteine mutant protein. (D) % bound fraction to heparin is compared between E3E2 WT and S154C+S296C mutant protein.

**Supplemental Table S1:** List of forward and reverse primers used for E3 gene synthesis through overlapping PCR method for making the E3 part of the E3E2 fusion protein and the primers used for amplifying E2 as well as E3E2 coding regions and also for creating E3E2 Lys/Arg to Ala point mutants for cloning into pET24b are listed above.

**Supplemental Table S2:** Results of Ellman’s assay on E3E2 cysteine mutants. DTNB (Ellman’s Reagent) (5,5-dithio-bis-(2-nitrobenzoic acid) (200 μM) was incubated with 0.1 mg/ml (2 μM) of E3E2 double cysteine mutant (S154C+S296C) and absorbance recorded at 412 nm. E3E2 wildtype protein is used as control. Increase in DTNB absorbance at 412 nm, compared to buffer blank, is seen only with double cysteine mutant protein, but not with wild type and mutant after fluorophore pair labelling. Assay was done with proteins in TNE pH 7.6 buffer.

## Abbreviations

CD: circular dichroism
CHIKV: chikungunya virus
EEEV: Eastern equine encephalitis virus
FL: fusion loop
FRET: fluorescence resonance energy transfer
GAG: glycosaminoglycans
HS: heparan sulfate
MD: molecular dynamics
NPT: constant Number of particles, Pressure and Temperature
NVT: constant Number of particles, Volume and Temperature
RRV: Ross River virus
SFV: Semliki Forest virus
SINV: Sindbis virus
SPC: Simple Point Charge
TIM1: T-cell immunoglobulin mucin domain 1
TNE: TN buffer containing 1 mM EDTA
