# Supplementary material for: Conformational changes in Chikungunya virus E2 protein upon heparan sulfate receptor binding explain mechanism of E2–E1 dissociation during viral entry
Source: Biosci Rep. 2019 Jun 28;39(6):BSR20191077. doi: 10.1042/BSR20191077 (PMC6597851; doi:10.1042/BSR20191077)

**Conformational changes in Chikungunya virus E2 protein upon heparan sulfate receptor binding explain mechanism of E2-E1 dissociation during viral entry**

Bibekananda Sahoo [1] and Tirumala Kumar Chowdary [1]*

**Table S1:** List of forward and reverse primers used for E3 gene synthesis through overlapping PCR method for making the E3 part of the E3E2 fusion protein and the primers used for amplifying E2 as well as E3E2 coding regions and also for creating E3E2 Lys/Arg to Ala point mutants for cloning into pET24b are listed above.

| Sl. No. | Primer name | Primer sequence | Remarks |
|---|---|---|---|
| 1 | **E3-RP0** | GCAAGACTTCTAGAATAC | **Primers used for E3 CDS synthesis using assembly PCR method** |
| 2 | **E3-FP0** | GTATTCTAGAAGTCTTGCCATCCCAGTTATGTG | |
| 3 | **E3-RP18** | ATTTGCCAACAGGCACATAACTGGGATG | |
| 4 | **E3-FP33** | CCTGTTGGCAAATACCACGTTCCCC | |
| 5 | **E3-RP46** | GGCTGGGAGCAGGGGAACGTGGT | |
| 6 | **E3-FP58** | TGCTCCCAGCCCCCTTGCACGC | |
| 7 | **E3-RP69** | CGTAGCAGCAGGGCGTGCAAGGG | |
| 8 | **E3-FP80** | CCTGCTGCTACGAAAAGGAACCGGA | |
| 9 | **E3-RP92** | GCGTAGGGTTTCCTCCGGTTCCTTTT | |
| 10 | **E3-FP105** | GGAAACCCTACGCATGCTTGAGGACA | |
| 11 | **E3-RP118** | GGTCTCATGACGTTGTCCTCAAGCAT | |
| 12 | **E3-FP131** | ACGTCATGAGACCTGGGTACTATCAGC | |
| 13 | **E3-RP144** | GGATGCTTGTAGCAGCTGATAGTACCCA | |
| 14 | **E3-FP158** | TGCTACAAGCATCCTTAACATGTTCTCCC | |
| 15 | **E3-RP172** | GCTGGCGGTGGGGAGAACATGTTAA | |
| 16 | **E3-FP187** | CACCGCCAGCGACGCAGCAGATCTAG | |
| 17 | **E3-RP197** | CTAGATCTGCTGCGTC | |
| 18 | **E3-FP** | GCTCATATGAGTCTTGCCATCCCAGTTATG | **E3-FP with NdeI site** |
| 19 | **E3-RP** | CTAGATCTGCTGCGTC | **E3-RP with BglII site for fusing with E2 start** |

| | | | |
|---|---|---|---|
| **20** | **E2-FP1** | AGCATATGACCAAGGACAACTTCAATGTCTATA | **E2-FP with NdeI site** |
| **21** | **E2-FP2** | AGAGATCTACCAAGGACAACTTCAATGTCTATA | **E2-FP with BglII site for fusing with E3 end** |
| **22** | **E2-RP** | ATGCGGCCGCGTACAGCTCATAATAATACA | **E2-RP with NotI site** |
| **23** | **E3E2-FP** | GCTCATATGAGTCTTGCCATCCCAGTTATG | **E3E2-FP with NdeI site** |
| **24** | **E3E2-RP** | ATGCGGCCGCGTACAGCTCATAATAATACA | **E3E2RP with NotI site** |
| **25** | **E2/R104A-FP** | CACTTCATCCTGGCCGCTTGTCCAAAAGGGGAA | **FP for incorporating R104A mutation into E3E2** |
| **26** | **E2/R104A-RP** | TTCCCCTTTTGGACAAGCGGCCAGGATGAAGTG | **RP for incorporating R104A mutation into E3E2** |
| **27** | **E2/K107A-FP** | CTGGCCCGATGTCCAGCAGGGGAAACTCTGACG | **FP for incorporating K107A mutation into E3E2** |
| **28** | **E2/R107A-RP** | CGTCAGAGTTTCCCCTGCTGGACATCGGGCCAG | **RP for incorporating K107A mutation into E3E2** |
| **29** | **E2/R104A+K107A-FP** | CACTTCATCCTGGCCGCTTGTCCAGCAGGGGAAACTCTGACG | **FP for incorporating both R104A and K107A mutations into E3E2** |
| **30** | **E2/R104A+K107A-RP** | CGTCAGAGTTTCCCCTGCTGGACAAGCGGCCAGGATGAAGTG | **RP for incorporating both R104A and K107A mutations into E3E2** |
| **31** | **E2/S154C-FP** | AAAGAGCTACCTTGCTGCACGTACGTGCAGA | **FP for incorporating S154C mutation into E3E2** |
| **32** | **E2/S154C-RP** | TCTGCACGTACGTGCAGCAAGGTAGCTCTTT | **RP for incorporating S154C mutation into E3E2** |
| **33** | **E2/S296C-FP** | ACCCAACACTCCTGTGCTACCGGAATATGGG | **FP for incorporating S296C mutation into E3E2** |
| **34** | **E2/S296C-RP** | CCCATATTCCGGTAGCACAGGAGTGTTGGGT | **RP for incorporating S296C mutation into E3E2** |

**Table S2:** Results of Ellman's assay on E3E2 cysteine mutants. DTNB (Ellman's Reagent) (5,5-dithio-bis-(2-nitrobenzoic acid) (200 μM) was incubated with 0.1 mg/ml (2 μM) of E3E2 double cysteine mutant (S154C+S296C) and absorbance recorded at 412 nm. E3E2 wildtype protein is used as control. Increase in DTNB absorbance at 412 nm, compared to buffer blank, is seen only with double cysteine mutant protein, but not with wild type and mutant after fluorophore pair labelling. Assay was done with proteins in TNE pH 7.6 buffer.

| | **TNE buffer** | **E3E2-WT** | **Unlabeled E3E2-S154C+S296C** | **Labeled E3E2-S154C+S296C** |
|---|---|---|---|---|
| **Absorbance @412nM** | **0.14** | **0.14** | **0.24** | **0.14** |

**Figure S1:** Sequence of CHIKV E3E2 and E2 protein used in this study. Protein sequence of E3 and E2 protein from Chikungunya virus strain LR2006_OPY1 (extracted from full-length structural poly-protein sequence, protein_id="ABD95938.1")

```
        10         20         30         40         50         60
SLAIPVMCLL ANTTFPCSQP PCTPCCYEKE PEETLRMLED NVMRPGYYQL LQASLTCSPH

        70         80         90        100        110        120
RQRRSTKDNF NVYKATRPYL AHCPDCGEGH SCHSPVALER IRNEATDGTL KIQVSLQIGI

       130        140        150        160        170        180
KTDDSHDWTK LRYMDNHMPA DAERAGLFVR TSAPCTITGT MGHFILARCP KGETLTVGFT

       190        200        210        220        230        240
DSRKISHSCT HPFHHDPPVI GREKFHSRPQ HGKELPCSTY VQSTAATTEE IEVHMPPDTP

       250        260        270        280        290        300
DRTLMSQQSG NVKITVNGQT VRYKCNCGGS NEGLTTTDKV INNCKVDQCH AAVTNHKKWQ

       310        320        330        340        350        360
YNSPLVPRNA ELGDRKGKIH IPFPLANVTC RVPKARNPTV TYGKNQVIML LYPDHPTLLS

       370        380        390        400        410        420
YRNMGEEPNY QEEWVMHKKE VVLTVPTEGL EVTWGNNEPY KYWPQLSTNG TAHGHPHEII

       430        440        450        460        470        480
LYYYELYPTM TVVVVSVATF ILLSMVGMAA GMCMCARRRC ITPYELTPGA TVPFLLSLIC

CIRTAKA
```

**E3** – sequence shown in gray colored text

**E2 -** ectodomain aminoacid sequence shown in blue colored text, transmembrane region is in black colored text, cytoplasmic tail sequence is in orange colored text

**Bold black arrow** – marks end of E3 and beginning of E2 proteolytic cleavage site.

**Dashed black arrow** – marks end of E2 ectodomain and beginning of E2 transmembrane region

**Figure S2:** Characterization of E3E2 WT and HBD mutants, E3E2 R104A, K107A and E3E2 R104A+K107A, proteins. (A) Intrinsic tryptophan fluorescence spectra of wildtype and single or double mutant proteins. (B) far-UV CD spectra of wildtype and single or double mutant proteins. (C) SDS-PAGE gel images from the heparin affinity binding experiment are shown in a comparison between E3E2 WT and both single and double Ala mutant proteins.

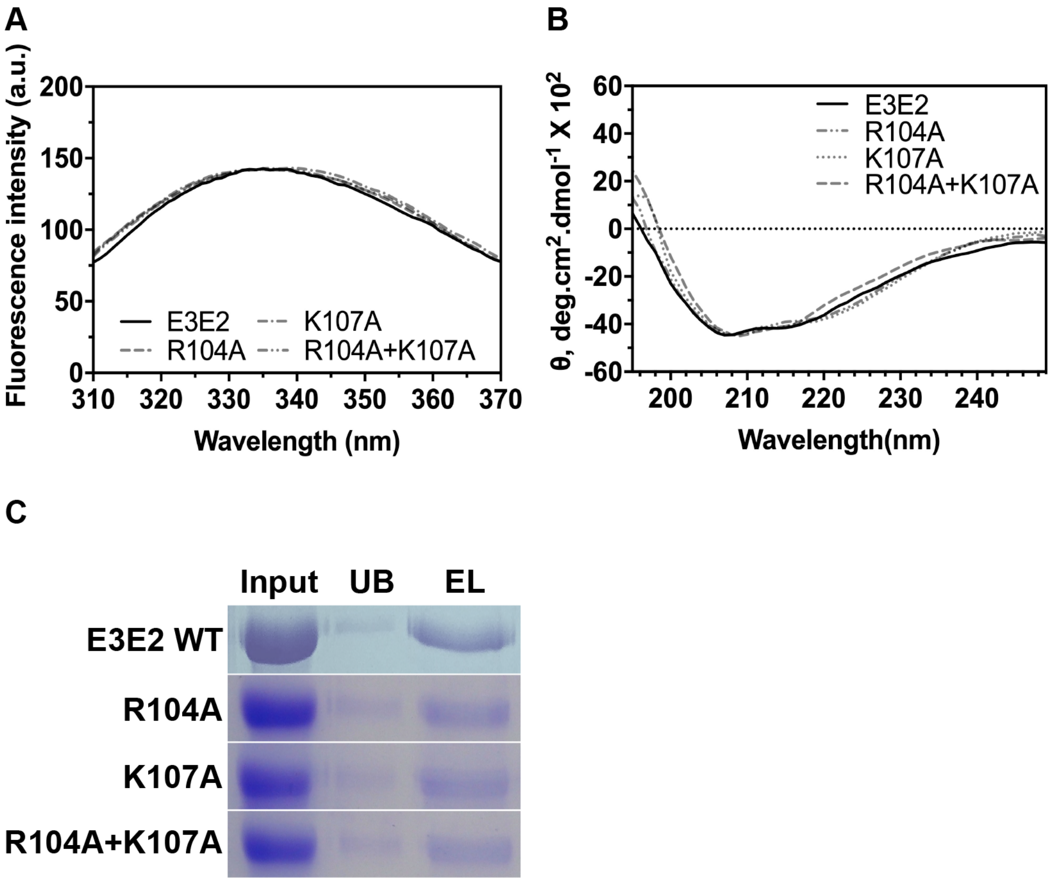

**Figure S3:** Characterization of E3E2 WT and S154C+S296C mutant protein. (A) Intrinsic tryptophan fluorescence spectra of wildtype and double cysteine mutant proteins. (B) far-UV CD spectra of wildtype and double cysteine mutant proteins. (C) SDS-PAGE gel images from the heparin affinity binding experiment are shown in a comparison between E3E2 WT and the double cysteine mutant protein. (D) % bound fraction to heparin is compared between E3E2 WT and S154C+S296C mutant protein.

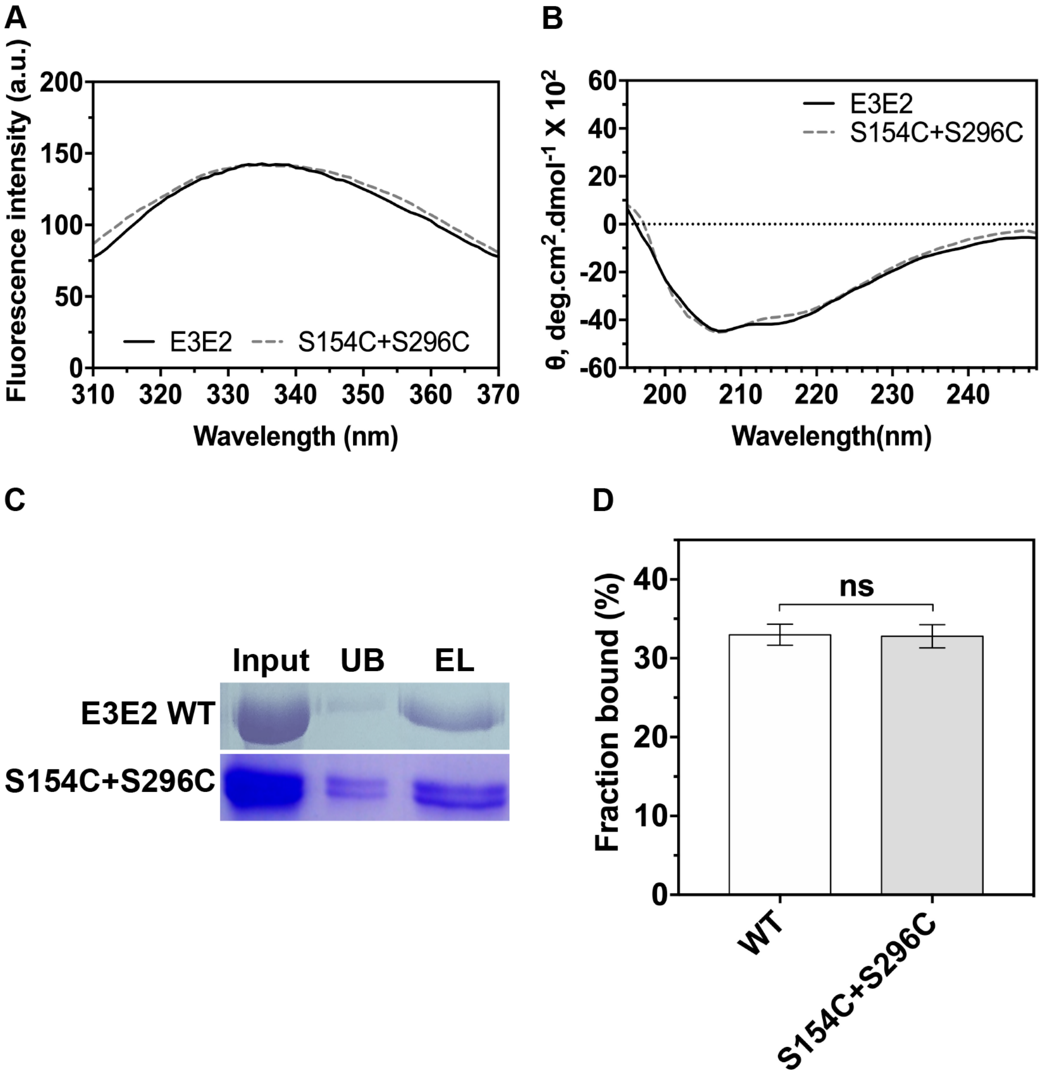

Supplement: Supplementary file 1 [file bsr20191077_Supp1.pdf]
